# Multi-Level Determinants of Food Insecurity among Racially and Ethnically Diverse College Students

**DOI:** 10.3390/nu15184065

**Published:** 2023-09-20

**Authors:** Nashira I. Brown, Acadia W. Buro, Rashida Jones, David Himmelgreen, Amber D. Dumford, Kyaien Conner, Marilyn Stern, Rita DeBate

**Affiliations:** 1 Department of Health Outcomes and Behaviors, Moffitt Cancer Center, Tampa, FL 33612, USA; nashira.brown@moffitt.org (N.I.B.); acadia.buro@moffitt.org (A.W.B.); 2 College of Population Health, University of New Mexico Health Sciences Center, Albuquerque, NM 87106, USA; 3 College of Public Health, University of South Florida, Tampa, FL 33612, USA; rashidajones@usf.edu; 4 Department of Anthropology, Center for the Advancement of Food Security and Healthy Communities, University of South Florida, Tampa, FL 33612, USA; dhimmelg@usf.edu; 5 College of Education, University of South Florida, Tampa, FL 33612, USA; dumford@usf.edu; 6 School of Social Work, University of Pittsburgh, Pittsburgh, PA 15260, USA; koconner@usf.edu; 7 Department of Child and Family Studies, College of Behavioral and Community Sciences, University of South Florida, Tampa, FL 33612, USA; mstern1@usf.edu

**Keywords:** food insecurity, disparities, college students

## Abstract

Compared with the general population, the prevalence of food insecurity (FI) is higher among college students. The COVID-19 pandemic exacerbated FI disparities and highlighted the need for further research to better understand and address FI in this population. Although race and ethnicity are two of the strongest predictors of FI among college students, little research is available on the determinants of FI among racial/ethnic minority college students. A cross-sectional study (*n* = 588) based on the National Institute of Minority Health and Health Disparities research framework was examined to identify population-specific determinants of FI among racially/ethnically diverse college students through the assessment of multiple domains (behavioral, environmental, socio-cultural) and levels of influence (individual, interpersonal, and community levels). Discrimination was the sole predictor of FI for non-Hispanic Black students. Coping mechanisms for FI (savings, reduced intake) and body mass index (BMI) were predictors of FI for Hispanic and non-Hispanic White students. Additionally, decreased holistic support from faculty and staff was also observed as a predictor of FI in Hispanic students. Implications include the need for further research and the development of multi-level, tailored interventions to address FI among college students with the goal of decreasing disparities.

## 1. Introduction

College students are more food insecure when compared to the general public [1,2,3]. Multi-university studies report 35–50% of students observed with food insecurity (FI) [4,5,6], with 14–59% observed via single-university prevalence studies [7,8,9]. FI is associated with many physical health issues [7,8,10] and has been correlated with mental health issues such as stress, anxiety, depression, and suicidal ideation [7,10]. Additionally, among college students, FI has been associated with lower academic performance [1,2,11,12,13], often affecting the likelihood of graduation [14], thus contributing to disparities in social determinants (i.e., economic stability, education access and quality) of health [15,16,17].

### Food Insecurity among Racially and Ethnically Diverse College Students

Underserved and underrepresented students are at greater risk of FI [1,4,7,10]. African American and Hispanic/Latino college students are 1.5× more likely to be food insecure compared to their non-Hispanic White and Asian counterparts [3,10,18]. In a recent secondary analysis of a single university’s National College Health Assessment 2020 data, 44.5% of students reported FI. Statistically significant differences were observed in FI by race/ethnicity (*p* < 0.001); a greater percentage of students who self-identified as Black/African American (57.7%) or Hispanic/Latino (47.9%) were food insecure as compared to students who self-identified as White (40.3%) [19]. Moreover, Black/African American students experiencing FI were observed with reduced flourishing (i.e., thriving) scores (*p* = 0.031), while Hispanic/Latino students experiencing FI were observed with higher psychological distress (*p* < 0.001), greater loneliness (*p* = 0.036), and lower resilience (*p* = 0.032) as compared to those who were food secure [19].

FI determinants include housing insecurity [20]; living off campus [8,13,18]; working ≥ 20 h per week [3,7]; household income ≤ $20 K [3,7,8]; year of study [8]; government support [21,22,23]; not budgeting [21]; and being a single parent [24]. Yet, race and ethnicity have been noted as the two of the strongest predictors of FI among college students [25], with racial discrimination associated with increased odds of experiencing FI [26,27]. The COVID-19 pandemic has highlighted and exacerbated FI disparities [28]. While the U.S. experienced a 60% increase in FI during the height of the COVID-19 pandemic, African Americans experienced an 80% increase, further widening existing disparities [29].

FI has been classified as a leading health indicator by Healthy People 2030, with the following call to action: “Reduce household FI and in doing so reduce hunger [14]”. Despite its public health significance, little research is available that explores the factors necessary to increase our understanding of FI among racial/ethnic minority college students. Addressing FI among racially/ethnically diverse college students requires an examination of determinants that span various domains within and among socio-ecological levels of influence to inform the development of interventions aimed at decreasing FI disparities [11,30]. The National Institute of Minority Health and Health Disparities (NIMHD) research framework offers a multi-dimensional model of multiple domains (behavioral, environmental, socio-cultural) and levels of influence (individual, interpersonal, and community levels) in understanding and addressing health disparities among racial/ethnic minority populations [31]. To this end, this study aims to explore FI among racially and ethnically diverse college students through a multi-dimensional lens with the purpose of identifying population-specific determinants of FI among racially/ethnically diverse college students.

## 2. Materials and Methods

### 2.1. Study Design, Setting, and Population

This cross-sectional study was conducted among college students attending a large, urban public university in the Southeast and was approved by the Institutional Review Board (IRB # 004835).

A list of all students meeting the following eligibility criteria was obtained from the university registrar: (1) currently enrolled as an undergraduate student and (2) 18 years old or older. There were 26,751 students meeting our study’s eligibility criteria. From this pool, we utilized stratified random sampling to select 25% of non-Hispanic White students, 100% of Hispanic/Latino/a students, and 100% of non-Hispanic Black/African American students. These strata and percentages were chosen to maximize the number of students of color in our sample and allow us to run the study’s planned analytical models. The stratified random sampling resulted in 15,528 students, who were emailed a link to the study screener survey. Individuals responding positively to a one-item FI screener (In the last 30 days, did you ever cut the size of your meals or skip meals because there wasn’t enough money for food?) [32] were invited to participate in the study. Following the invitation to participate in the study, participants provided informed consent and were emailed a link to the electronic survey housed in the Research Electronic Data Capture (REDCap) web-based application [33,34]. Upon completion, participants received a USD 25 Amazon Gift Card. Of the 15,528 that were emailed the screener, 1443 (9.3%) responded and completed the eligibility screening. A total of 860 (59.6%) students were invited to participate, 804 (93.5%) consented, and 685 (85.2%) completed the survey (non-Hispanic Black (*n* = 203), Hispanic/Latino/a (*n* = 357); non-Hispanic White (*n* = 125). A total of 97 responses were removed due to missing data, with the final sample including 588 participants. 

#### Conceptual Framework

The National Institute on Minority Health and Health Disparities (NIMHD) research framework [31] served as the conceptual framework for the current study. The NIMHD research framework serves as a vehicle in encouraging research that addresses the multi-faceted nature of minority health that spans multiple domains of influence (i.e., health outcomes, behaviors, environment, socio-cultural environment) within multiple levels of influence (i.e., individual, interpersonal, community) [31].

### 2.2. Measures

A web-based survey was developed to assess domain-specific determinants of FI among racially/ethnically diverse college students across multiple levels of influence based on the NIMHD research framework [31]. Constructs assessed by level of influence and domain were as follows. (a) Individual level of influence: health domain—food insecurity [35]; psychological distress [36]; loneliness [37]. (b) Individual level of influence: behavioral domain—food insecurity coping and academic progress [38,39]; cooking behaviors and skills [38,39]. (c) Individual level of influence: socio-cultural domain—sociodemographics and cultural identity. (d) Interpersonal level of influence: socio-cultural environmental domain—experience of discrimination [40]; social support (family, friends) [41]. (e) Community level of influence: socio-cultural environmental domain—cultural familiarity, cultural validation, humanized educational experience, holistic support [42]. Instruments employed within levels/domains are presented in Table 1.

#### 2.2.1. Individual Level of Influence Measures: Health Domain

Food Insecurity. The USDA Household Food Security Short Form [35] is a validated, six-item scale that assesses household food insecurity and hunger in the last 12 months. Increased affirmative responses to items (e.g., “*The food that (I/we) bought just didn’t last, and (I/we) didn’t have money to get more.*” *Was that often, sometimes, or never true for (you/your household) in the last 12 months?*) indicate higher food insecurity levels. Possible food security scores range from 0 to 6, with higher scores indicating lower levels of food security (i.e., FI). Scores were categorized as 0–1 = marginal food security; 2–4 = low food security; 5–6 = very low food security. This measure has demonstrated validity in food insecurity among college students [7] and acceptable reliability (Cronbach α = 0.87) [43].

Psychological Distress. The Kessler Psychological Distress scale (K6, 6 items) [36] measures psychological distress by asking how frequently the respondent experienced symptoms of psychological distress (e.g., nervous, hopeless) during the past 30 days. Responses to the items are assessed on a 5-point Likert scale (0 = none of the time to 4 = all the time) and summed to yield a total score ranging from 0 to 24. Higher scores are indicative of high levels of psychological distress. This scale has demonstrated acceptable reliability (Cronbach α = 0.89) [36].

Loneliness. The UCLA Loneliness Scale [37] is a 3-item measure that assesses the lack of companionship, feeling left out, and feeling isolated from others on a 3-point Likert scale (1 = hardly ever to 3 = often). A total score is generated, ranging from 3 to 9, with higher scores indicating higher levels of loneliness. This scale has shown acceptable internal reliability (Cronbach α = 0.72) [37]. 

#### 2.2.2. Individual Level of Influence Measures: Behavioral Domain

Coping Mechanisms Food Insecurity. The coping strategies scale (CSS, 29 items) [38,39] measures how often students use coping strategies (i.e., saving, food intake/access, and selling items to be able to buy food) to address FI. Responses are scored using a 3-point Likert scale (1 = never to 3 = often). The possible scores for saving (9 items) range from 0 to 38, with higher scores indicating more saving strategies (e.g., took fewer classes, used less utilities, cut out food coupons, attended functions where there was free food). The possible scoring range for food intake/access (6 items) is 0 to 12, with higher scores indicating more reduced food intake strategies (e.g., ate more than needed when food was plentiful, purchased cheap, processed foods, bartered items/services to buy food). The scores for selling items (4 items) range from 0 to 8, with higher scores demonstrating more selling of things for food strategies (sold textbook, personal possessions, blood/plasma, sperm/eggs to buy food). The overall total possible CSS score ranges from 29 to 87, with higher summed scores indicating more use of coping strategies in response to FI. 

School Functioning. The Academic Progress Scale (APS, 4 items) [38,39] assesses students’ perceived academic behaviors in relation to class attendance using a 4-point Likert scale (1 = poor to 4 = excellent). APS scores range from 4 to 16, with higher summed scores corresponding to better perceived academic behaviors. 

Cooking Related. There were two food-related items. First, the frequency of cooking for self and others [38] was assessed using a one-item, 3-point Likert scale (0 = never to 3 = often). A higher score indicates a higher frequency of cooking for self or others. Second, perceived cooking skills [38] were scored using a one-item, 4-point Likert scale (1 = poor to 4 = excellent). A higher score indicates higher perceived cooking skills. 

#### 2.2.3. Individual Level of Influence: Socio-Cultural Domain

Sociodemographics. Participant characteristics (15 items) included age, height, weight, health status, undergraduate level (freshman, sophomore, junior, senior), enrollment status (full-time vs. part-time), employment status (working < 20 h/week, working > 20 h/week, not working), sources of financial support (loans, grants/scholarships, working, and/or parents/family), having a Pell Grant, meal plan, and/or currently have/ever had a physical or learning disability, relationship status (single, dating, girlfriend/boyfriend, married/partnered, divorced/annulled). Body mass index (BMI) was calculated using self-reported weight and height (weight (kg)/[height (m)]^2^). Living situation characterization was assessed with two items. Students were asked where they currently lived (campus/university, parent/guardian, off-campus, couch surfing, don’t have a place) and with whom they lived (roommates, significant other, family, self). 

Cultural Identity. Five survey questions captured students’ gender identity (woman/female, man/male, trans man, genderqueer, agender, genderfluid, non-binary), sexual orientation (straight/heterosexual, bisexual, gay, lesbian, queer, questioning, other, prefer not to respond), sex assigned at birth (female, male, intersex), race (White, Black/African American), and ethnicity (Hispanic vs. non-Hispanic). 

#### 2.2.4. Interpersonal Level of Influence Measures: Socio-Cultural Environmental Domain 

Experiences of Discrimination. The Experiences of Discrimination Scale assessed self-reported experiences of discrimination (Cronbach α = 0.74 or greater) [40]. The Discrimination Ever subscale [40] is a 9-item measure that assesses whether an individual has ever experienced discrimination in nine situations (e.g., at school, at work, getting service in a store) due to their race, ethnicity, or color. A total score is calculated ranging from 0 to 9, with higher scores indicating greater instances of ever experiencing discrimination. The Day-to-Day Unfair Treatment sub-scale measures the frequency of self-reported experiences of discrimination in everyday life (e.g., treated with less courtesy, less respect, people acted as if they are afraid of you, you have been called names) using a 10-item, 4-point Likert scale (1 = four or more times to 4 = never). A total sum score is calculated, with higher scores indicating higher discrimination in everyday situations. The Major Experiences of Discrimination scale is a 9-item measure that assesses whether an individual has experienced unfair treatment (i.e., unfairly fired, unfairly stopped, unfairly discouraged by a teacher). A sum score is calculated to reflect the number of situations in which an individual has experienced unfair treatment in relation to a racial reason with possible scores ranging from 0 to 9, with higher scores indicating greater instances of experiencing unfair treatment.

Social Network. The Lubben Social Network Scale [41] is a 12-item measure of social engagement from family (6 items) and friends (6 items). This instrument uses a 6-point Likert scale (0 = less social engagement to 5 = more social engagement) with a total score calculated as the sum of all items. The total score can range between 0 and 30 for each of the two social support scales (family and friends), with higher scores indicating more social engagement. These scales have demonstrated acceptable internal reliability (Cronbach α = 0.84–0.89 for family and 0.80–0.82 for friends) [44].

#### 2.2.5. Community Level of Influence Measures: Socio-Cultural Environmental Domain

Cultural familiarity, validation, humanized environment, holistic support. The Culturally Engaging Campus Environments (CECE) is a scale that measures campus environments and student experiences using a 5-point Likert scale (1 = strongly agree to 5 = strongly disagree) [42,45]. This scale consists of two subconstructs of cultural relevance (cultural familiarity and cultural validation) and two subconstructs of cultural responsiveness (humanized educational environment and holistic support). Cultural familiarity is composed of 3 items that measure students’ opportunities to connect with various agents on campus (e.g., faculty administrators, staff, and peers) who are like them in terms of background and experiences. Responses from this scale are summed. The total score ranges from 5 to 25, with higher scores indicating a campus environment with greater cultural familiarity. This scale has been shown to have strong reliability in past studies (Cronbach α = 0.87) [42]. Cultural validation, or campus cultures that validate the cultural backgrounds, knowledge, and identities of diverse students, was measured with 3 items. Possible scores ranged from 3 to 15, with higher scores indicating a campus environment with greater cultural validation. Strong reliability was indicated for this scale (Cronbach α = 0.92) [45]. Humanized Educational Environment, or the availability of opportunities for students to develop meaningful relations with members of faculty and staff who care about and are committed their success, was assessed with 3 items. Responses to items were summed, with possible scores ranging from 3 to 15. Higher scores indicate a campus environment with greater humanized educational experiences. This scale has shown strong reliability (Cronbach α = 0.92) [45]. Holistic support measured student’s access to at least one faculty or staff member in whom the student was confident that they could provide information that they need, offer the help they seek, or connect them with the information or support that they require regardless of the problem or use that they face, with 4 items. Responses are summed, with a total possible score ranging from 4 to 20, with higher scores reflecting a campus environment with greater holistic support. This scale has demonstrated strong reliability (0.90) [45].

### 2.3. Statistical Analysis

First, descriptive statistics were used to summarize participant characteristics and compare survey responses across race/ethnicity. FI and multi-dimensional, NIMHD-based constructs were analyzed as continuous variables (sum scores). Associations between FI and constructs were analyzed using Pearson correlation coefficients. The differences in construct scores by race/ethnicity were analyzed using one-way analyses of variance (ANOVAs), followed by Tukey’s post-hoc tests.

In the next step, four linear regression models (one for all students and then each of the three subgroups: non-Hispanic White students; Hispanic/Latino/a students; non-Hispanic Black/African American) were conducted, with the level of FI as the dependent variable and NIMHD-based determinates (see Table 1; described in the Measures section) as independent variables. FI was measured as a continuous variable (sum score). For the purposes of this analysis, sources of financial support (parent/family, loans, grants/scholarships, and working) and living situation (on campus, parent/guardian, off campus, couch surfing) were collapsed into four separate dichotomous variables (all, 0 = no and 1 = yes). Health status was collapsed into a dichotomous variable with 0 = good or excellent and 1 = poor or fair. SPSS version 29 (IBM SPSS Inc., Armonk, NY, USA) was used to perform statistical analyses. *p*-values less than 0.05 were considered statistically significant.

## 3. Results

### 3.1. Population Characteristics

Respondents were White (65.8%), Hispanic (48.6%), Black/African American (34.2%), female (71.6%), full-time (90.6%), undergraduate seniors (33.5%), with an average age of 21 (SD = 4.1), financially supported by grants and/or scholarships (66.5%) and receiving federal Pell grants (65.1%). Table 2 presents the participant characteristics.

### 3.2. Associations between FI and Constructs

Pearson correlation coefficients are presented in Table 3. FI was significantly associated with all constructs (*p* < 0.05) except for social support from friends, frequency of cooking for self and others, and perceived cooking skills. The strongest relationships found were between food insecurity and FI coping strategy—saving (r = 0.367) and FI coping strategy—food intake (r = 0.344).

### 3.3. Differences in Construct Scores

As depicted in Table 4, although trending towards significance, no statistically significant differences were observed for FI by racial and ethnic group (*p* = 0.058). Nonetheless, ANOVAs revealed statistically significant differences in discrimination ever experience (ever experiencing discrimination), discrimination major (experienced unfair treatment), day-to-day unfair treatment (experiences of discrimination in everyday life), cultural familiarity (opportunities to connect with faculty administrators, staff, and peers with similar background and experiences), cultural validation (campus cultures that validate the cultural backgrounds, knowledge, and identities of diverse students), humanized education experience (availability of opportunities for students to develop meaningful relations with members of faculty and staff who care about and are committed to their success), social support from family, and social support from friends sum scores between racial and ethnic groups (Table 3). Post-hoc test results revealed statistically significant differences in cultural validation, humanized education experiences, and social support from family between non-Hispanic Black and Hispanic students (*p* < 0.001, *p* = 0.026, and *p* = 0.029, respectively). Specifically, non-Hispanic Black students had significantly lower scores than their Hispanic peers for cultural validation, humanized education experience, and social support from family. For discrimination ever experience and day-to-day unfair treatment sum scores, post-hoc analyses indicated that all group means were significantly different from one another (both, *p* < 0.001), with non-Hispanic Black students having statistically higher scores than Hispanic and non-Hispanic White students. The post-hoc test for discrimination major, cultural familiarity, and social support from friends indicated that non-Hispanic Black students were significantly different from both their Hispanic and non-Hispanic White peers (*p* < 0.001, *p* = 0.025, *p* = 0.011, respectively) but Hispanic students were not significantly different from non-Hispanic White students. Significantly higher scores of discrimination major were observed for non-Hispanic Black students when compared to Hispanic and non-Hispanic White students. Conversely, non-Hispanic Black students had significantly lower cultural familiarity and social support from friends when compared to their Hispanic and non-Hispanic White peers.

### 3.4. Determinants of FI

The associations found between all model determinants and FI from four separate regression models are shown in Table 5. The Variance Inflation Factor (VIF) values for each predictor variable in these regression models were all well below 5 (ranging from 1.0 to 2.3), suggesting that multicollinearity was not an issue in the models [46]. Model 1, consisting of all students (regardless of race or ethnicity), indicated that the Kessler6 sum scores, FI coping strategies (saving and food intake), Black race, BMI, and age were positively associated with FI (*p* = 0.007 *p* < 0.001, *p* = 0.002, *p* = 0.003, *p* = 0.009, and *p* = 0.026, respectively). Model 1 also indicated that working to financially support oneself was inversely associated with FI (*p* = 0.038). The two strongest predictors in the model were Black race (β = 0.301) and working to financially support oneself (β = −0.201). Model 2, including only non-Hispanic Black students, revealed only one statistically significant positive association between FI and discrimination major sum scores (β = 0.203; *p* < 0.001). Model 3, which included only Hispanic students, indicated a statistically significant positive association between FI and FI coping (saving and food intake) (*p* < 0.001 and *p* = 0.027, respectively) and BMI (*p* = 0.017). Model 3 also indicated a statistically significant, although rather small in magnitude, inverse relationship between FI and holistic support sum scores (β = −0.051; *p* = 0.007). Model 4, consisting of non-Hispanic White students, revealed a significant positive association between FI and FI coping (saving and food intake) (*p* < 0.001 and *p* = 0.012, respectively) and BMI (*p* = 0.019). No other determinants yielded any statistically significant associations with FI. The variance explained for each model is listed in Table 5. The predictor variables accounted for 7.8% to 32.3% of the total variance in the level of FI.

### 3.5. Summary of Main Findings

A summary of the study findings is presented in Table 6.

## 4. Discussion

Underserved and underrepresented students are at greater risk of FI and associated health and academic issues [1,4,7,10]. Addressing FI among racial/ethnic minority college students requires an examination of determinants that span various domains within and among socio-ecological levels of influence to inform the development of interventions aimed at decreasing FI disparities [11,30]. To our knowledge, this is the first known study to employ a multi-dimensional model to understand and address health disparities among a large group of racially and ethnically diverse college students [31]. Results from the study revealed three critical findings with regard to FI among college students. When examining potential predictors of FI among all college students, regardless of race and ethnicity, we found significant multi-level/domain determinants, some of which support previous work (i.e., coping mechanisms, age, BMI, race). Subsequently, the significant finding that race was a predictor revealed the need for separate models by race and ethnicity.

First, although the current study revealed no statistically significant differences between racial/ethnic groups regarding intrapersonal-level health and behavioral domains of influence, differences were observed within the interpersonal-level socio-cultural and the community-level socio-cultural domains. Within both levels and domains, the current study revealed that Non-Hispanic Black participants reported experiencing more discrimination and less social support (support from family and friends), cultural familiarity (opportunities to connect with faculty administrators, staff, and peers with similar background and experiences), cultural validation (campus cultures that validate the cultural backgrounds, knowledge, and identities of diverse students), and humanized educational experience (availability of opportunities for students to develop meaningful relations with members of faculty and staff who care about and are committed their success) as compared with their Hispanic and Non-Hispanic White counterparts. These findings are consistent with the literature on college students, with minority students reporting disproportionate discrimination. Specifically, Black/African American students experience a higher incidence of discrimination in comparison to non-Hispanic White individuals [47] and Hispanic individuals [48]. Non-Hispanic Black students had significantly lower cultural familiarity scores than both non-Hispanic White and Hispanic individuals. While the literature supports the lack of a sense of belonging and culture on college campuses among minority college students [49], it is unclear if this aspect affects food security.

Second, the current study revealed differences in multi-level determinants of FI between racially and ethnically diverse college students. When assessed collectively, our study supports the work of others in that working ≥ 20 h per week to financially support oneself [3,7] and race/ethnicity are among the strongest predictors of FI among college students [25]. These findings support the current literature and highlight the influence of social determinants on FI, especially among racial and ethnic minority college students. In a previous study, students who reported that they were employed were roughly two-times more likely to be food insecure [7], indicating that their income was not sufficient to meet their basic needs. Moreover, previous studies examining FI among college students have included food-secure and food-insecure students and have found significant differences in FI among racial and ethnic minority students [10]. Specifically, Black/African American and other racial/ethnic minority students are significantly more likely to be at risk of FI or be categorized as FI when compared to non-Hispanic White students [10,50]. Our findings support these previous studies, as Black race was found to be a statistically significant and the strongest predictor of food insecurity, when controlling for other factors and the other model determinants.

Third, perhaps one of the most important findings from the current study is the differences in multi-level determinants of FI by racial and ethnic student population. Differences were most stark between non-Hispanic Black students and their Hispanic and non-Hispanic White peers. For example, when analyzed separately, the single predictor of FI among non-Hispanic Black students was experiencing major discrimination. This finding supports the work of Burke et al., who observed an association between the frequency of lifetime racial discrimination and a very low level of food security [51]. In contrast, among Hispanic and Non-Hispanic White participants, determinants of FI included increased FI coping strategies (saving, intake) and increased BMI. While a previous study has reported that food-insecure students are more likely to use coping strategies [38,39], no other study, to our knowledge, has examined differences in the use of such strategies by race and ethnicity. Moreover, decreased holistic support was also revealed as a determinant of FI among Hispanic students. There is a paucity of existing literature on holistic support in relation to FI. However, Hagedorn-Hatfield et al. have emphasized the necessity to include such needs programs on campus to establish a nutritionally secure campus to alleviate insecurity and increase student success [52].

The current study has several strengths as it is the first to (a) address a major limitation in FI research among racial/ethnic minorities in a higher education community setting; (b) employ a multi-level and domain conceptual framework to assess FI among racial and ethnic college students with higher rates of FI and associated health issues for the assessment of determinants; and (c) identify population-specific determinants of FI among racial/ethnic minority college students through the assessment of various domains and levels of influence as noted in the NIMHD research framework [31]. However, the interpretation of findings should be considered within the study limitations. Our study sample consisted purely of undergraduate-level students at a single university, which affects its generalizability to graduate-level and college students attending other institutions (e.g., community colleges) in other geographical regions. Additionally, the reliance on self-reported experiences (e.g., discrimination) over a long duration (e.g., in the past 12 months) can potentially introduce recall bias and affect the validity of results. Further, self-reported participant characteristics (e.g., weight and height) can be inaccurate. Lastly, we did not discuss the finding of non-significant, yet trending (*p* < 0.058) differences between racial/ethnic groups regarding intrapersonal-level health and behavioral domains of influence, but clearly this warrants further exploration, perhaps with a larger sample of even more diverse students, at multiple universities.

## 5. Conclusions

This study provides a nuanced understanding regarding the multi-level determinants of FI among racially and ethnically diverse college students. The findings can be used to inform the development of multi-component interventions aimed at reducing FI disparities and addressing community-level socio-cultural determinants regarding discrimination (for non-Hispanic Black students in particular) and holistic support.

Despite these important findings, further research is warranted to gain a better understanding of the specific determinants of FI among racially and ethnically diverse college students. As food insecurity has been associated with negative health-related outcomes including an increased risk of obesity [53], other chronic diseases [54], and poor mental health [55], institutions across the U.S. are implementing supports and programs to address food insecurity among students. Beyond providing access to campus food pantries, efforts have been focused on providing students with culturally responsive information about additional resources, such as Supplemental Nutrition Assistance Program (SNAP) benefits [56]. However, further efforts that address multi-level determinants are necessary as obstacles arise for students.

Findings of discrimination indicate a need for (1) qualitative research to gather rich data on students’ lived experiences, (2) multi-level culturally appropriate interventions developed in collaboration with Black students, and (3) the investigation of additional multi-level determinants, including relevant policies.

## Figures and Tables

**Table 1:** Multi-level determinants of FI based on NIHMD framework.

		Levels of Influence		
		Individual	Interpersonal	Community
Domains of Influence	Health	Food Insecurity Psychological Distress Loneliness		
	Behavioral	Food Insecurity Coping: Saving, Intake, Selling Cooking; Cooking Skills Employment	School Functioning Relationship Status	Cultural Familiarity Cultural Validation Humanized Educational Experience Holistic Support
	Physical/Built Environment	Housing Meal Plan	Living Situation	
	Socio-Cultural Environment	Sociodemographic Cultural Identity	Experience of Discrimination: Day-to-Day Unfair Treatment Social Support: Family, Friends	Experience of Discrimination: Ever, Major Experiences

**Table 2:** Participant demographics for a sample of college students by race/ethnicity (*n* = 588).

	Non-Hispanic Black	Hispanic	Non-Hispanic White	Total
	M (SD)	M (SD)	M (SD)	M (SD)
Age	21.1 (4.2)	20.7 (3.1)	21.7 (5.5)	21.1 (4.1)
Weight (lbs.)	164.3 (43.3)	152.3 (38.5)	153.9 (34.9)	156.2 (39.6)
Height (inches)	66.4 (4.0)	65.3 (3.6)	66.6 (4.0)	65.9 (3.9)
BMI (kg/m^2^)	26.1 (6.2)	25.0 (5.3)	24.3 (4.6)	25.2 (5.5)
	*n* (%)	*n* (%)	*n* (%)	*n* (%)
Race				
White	0 (0.0)	260 (90.9)	127 (100.0)	387 (65.8)
Black	175 (100.0)	26 (9.1)	0 (0.0)	201 (34.2)
Ethnicity				
Hispanic	0 (0.0)	286 (100.0)	0 (0.0)	286 (48.6)
Not Hispanic	175 (100.0)	0 (0.0)	127 (100.0)	302 (51.4)
Sex (assigned at birth)				
Female	127 (72.6)	206 (72.0)	88 (69.3)	421 (71.6)
Male	47 (26.9)	80 (28.0)	39 (30.7)	166 (28.2)
Intersex	1 (0.6)	0 (0.0)	0 (0.0)	1 (0.2)
Gender identity				
Woman/female	127 (72.6)	197 (68.9)	80 (63.0)	404 (68.7)
Man/male	45 (25.7)	79 (27.6)	39 (30.7)	163 (27.7)
Trans man	0 (0.0)	0 (0.0)	1 (0.8)	1 (0.2)
Genderqueer	1 (0.6)	1 (0.3)	2 (1.6)	4 (0.7)
Agender	0 (0.0)	1 (0.3)	0 (0.0)	1 (0.2)
Genderfluid	1 (0.6)	3 (1.0)	2 (1.6)	6 (1.0)
Non-binary	1 (0.6)	5 (1.7)	3 (2.4)	9 (1.5)
Sexual orientation				
Straight/heterosexual	137 (78.3)	209 (73.1)	86 (67.7)	432 (73.4)
Bisexual	16 (9.1)	42 (14.7)	29 (22.8)	87 (14.8)
Gay	5 (2.9)	3 (1.1)	0 (0.0)	8 (1.4)
Lesbian	2 (1.1)	9 (3.1)	4 (3.1)	15 (2.6)
Queer	5 (2.9)	8 (2.8)	3 (2.4)	16 (2.7)
Questioning	3 (1.7)	5 (1.7)	0 (0.0)	8 (1.4)
Other	5 (2.9)	2 (0.7)	3 (2.4)	10 (1.7)
Prefer not to respond	2 (1.1)	8 (2.8)	2 (1.6)	12 (2.0)
Student status				
Full-time	158 (90.3)	257 (89.9)	118 (92.9)	533 (90.6)
Part-time	17 (9.7)	27 (9.4)	9 (7.1)	53 (9.0)
Undergraduate level				
Freshman	26 (14.9)	43 (15.0)	24 (18.9)	93 (15.8)
Sophomore	35 (20.0)	59 (20.6)	21 (16.5)	115 (19.6)
Junior	57 (32.6)	89 (31.1)	37 (29.1)	183 (31.1)
Senior	57 (32.6)	95 (33.2)	45 (35.4)	197 (33.5)
Housing				
Campus/university	54 (30.9)	63 (22.0)	31 (24.4)	148 (25.2)
Parent/guardian	35 (20.0)	91 (31.8)	22 (17.3)	148 (25.2)
Off-campus	82 (46.9)	131 (45.8)	70 (55.1)	283 (48.1)
Couch surfing	4 (2.3)	1 (0.3)	4 (3.1)	9 (1.5)
Don’t have a place	0 (0.0)	0 (0.0)	0 (0.0)	0 (0.0)
Living situation				
Roommates	117 (67.0)	163 (57.0)	82 (64.6)	362 (61.6)
Significant other	4 (2.3)	19 (6.6)	14 (11.0)	37 (6.3)
Family	42 (24.0)	99 (34.6)	26 (20.5)	167 (28.4)
By myself	11 (6.3)	5 (1.7)	5 (3.9)	21 (3.6)
Relationship status				
Single	120 (68.6)	135 (47.2)	51 (40.2)	306 (52.0)
Dating	14 (8.0)	27 (9.4)	16 (12.6)	57 (9.7)
Girlfriend/boyfriend	39 (22.3)	114 (39.9)	52 (40.9)	205 (34.9)
Married/partnered	2 (1.1)	10 (3.5)	5 (3.9)	17 (2.9)
Divorced/annulled	0 (0.0)	0 (0.0)	3 (2.4)	3 (0.5)
Employment status				
Yes, <20 h/week	68 (38.9)	109 (38.1)	42 (33.1)	219 (37.2)
Yes, >20 h/week	41 (23.4)	65 (22.7)	42 (33.1)	148 (25.2)
No	65 (37.1)	112 (39.2)	42 (33.1)	219 (37.2)
Financial Support ^a^				
Parents/family	106 (60.6)	173 (60.5)	63 (49.6)	342 (58.2)
Loans	62 (35.4)	71 (24.8)	44 (34.6)	177 (30.1)
Grants/scholarships	117 (66.9)	192 (67.1)	82 (64.6)	391 (66.5)
Working	106 (60.6)	177 (61.9)	86 (67.7)	369 (61.5)
Federal Pell Grant				
Yes	114 (65.1)	133 (46.5)	44 (34.6)	291 (49.5)
Meal plan				
Yes	49 (28)	71 (24.9)	34 (26.8)	154 (26.2)
Physical/learning disability				
Yes	13 (7.4)	31 (10.8)	24 (18.9)	68 (11.6)

Note: ^a^ Participants could choose more than one source of financial support. Abbreviations: BMI, body mass index.

**Table 3:** Pearson correlation matrix for food insecurity and multi-level constructs in a sample of racially/ethnically diverse college students (*n* = 588).

	FI
Psychological Distress	0.211 *
Loneliness	0.102 **
FI Coping Saving	0.367 **
FI Coping Intake	0.344 **
FI Coping Selling	0.168 **
Day-to-Day Unfair Treatment	0.156 **
Discrimination Major	0.182 **
Discrimination Everyday	0.189 **
Cultural Familiarity	−0.121**
Cultural Validation	−0.109 **
Humanized Educational Experience	−0.104 *
Holistic Support	−0.095 *
Academic Progress	−0.102 *
Social Support Family	−0.149 **
Social Support Friends	−0.069
Frequency of Cooking for Self and Others	0.058
Perceived Cooking Skills	0–0.010

Note: ** Correlation is significant at the 0.01 level (2-tailed). * Correlation is significant at the 0.05 level (2-tailed). Abbreviations: FI, food insecurity.

**Table 4:** Differences in food insecurity and multi-level construct scores for a sample of college students by race/ethnicity (*n* = 588) based on one-way analyses of variance (ANOVAs) and Tukey’s post-hoc tests.

	Non-Hispanic Black	Hispanic	Non-Hispanic White	Total	*p*-Value
Sum Scores	M (SD)	M (SD)	M (SD)	M (SD)	
Food Insecurity	4.9 (1.1)	4.7 (1.2)	4.6 (1.2)	4.7 (1.2)	0.058
Distress	9.7 (4.5)	10.1 (4.7)	10.1 (4.7)	10 (4.7)	0.606
Loneliness	6.1 (1.9)	5.8 (1.7)	5.9 (1.8)	5.8 (1.8)	0.094
FI Coping Scale (Saving)	12.7 (5.2)	13.1 (5.5)	12.9 (5.3)	12.9 (5.4)	0.752
FI Coping (Intake)	4.3 (2.2)	4.0 (2.0)	4.3 (2)	4.2 (2.1)	0.415
FI Coping (Selling)	0.47 (0.84)	0.54 (0.96)	0.57 (1)	0.52 (0.93)	0.666
Discrimination Ever Experience	3.3 (2.3)	1.7 (2.0)	0.83 (1.7)	2.0 (2.0)	<0.001 *
Discrimination Major	1.5 (1.5)	0.83 (1.2)	0.56 (1.0)	0.98 (1.3)	<0.001 *
Day-to-Day Unfair Treatment	14.6 (7.8)	8.9 (6.8)	4.9 (6.7)	9.7 (7.9)	<0.001 *
Cultural Familiarity	15.8 (5.0)	17.0 (4.4)	17.4 (4.8)	16.7 (4.7)	0.007 *
Cultural Validation	9.6 (3)	10.6 (2.7)	10.1 (3.2)	10.2 (2.9)	<0.001 *
Humanized Education Experience	10.5 (2.9)	11.2 (2.8)	11.1 (2.9)	11.0 (2.9)	0.029 *
Holistic Support	12.7 (3.6)	13.1 (3.5)	13.4 (3.6)	13.0 (3.6)	0.244
Academic Progress	11.7 (2.6)	12.1 (2.4)	11.9 (2.4)	12.0 (2.4)	0.259
Social Support Family	9.5 (3.7)	10.4 (3.9)	10.3 (3.8)	10.1 (3.8)	0.039 *
Social Support Friends	9.6 (3.8)	10.7 (3.6)	11.1 (3.9)	10.4 (3.8)	<0.001 *

Note: * Significant at the *p* < 0.05 level.

**Table 5:** Linear regression analyses of food insecurity and multi-level determinants among college students (*n* = 588).

	Model 1		Model 2		Model 3		Model 4	
	All Students		Non-Hispanic Black		Hispanic		Non-Hispanic White	
Predictor	β	*p*-Value	β	*p*-Value	β	*p*-Value	β	*p*-Value
Psychological Distress Sum Score	**0.028**	**0.007**	0.018	0.818	0.103	0.084	0.060	0.500
Loneliness Sum Score	0.001	0.985	−0.015	0.850	0.026	0.647	0.055	0.510
FI Coping (Saving) Sum Score	**0.063**	**<0.001**	0.09	0.250	**0.074**	**<0.001**	**0.102**	**<0.001**
FI Coping (Intake) Sum Score	**0.084**	**0.002**	0.164	0.063	**0.084**	**0.027**	**0.136**	**0.012**
FI Coping (Selling) Sum Score	0.043	0.0304	0.083	0.314	0.010	0.864	0.103	0.244
Discrimination Ever Experience Sum Score	0.035	0.431	0.109	0.272	0.046	0.416	−0.029	0.724
Discrimination Major Sum Score	0.058	0.181	**0.203**	**<0.001**	0.075	0.185	−0.046	0.575
Day-to-Day Unfair Treatment	0.047	0.300	0.146	0.107	0.039	0.483	−0.002	0.982
Cultural Familiarity Sum Score	−0.035	0.392	0.041	0.620	−0.048	0.425	−0.014	0.860
Cultural Validation Sum Score	−0.026	0.517	0.002	0.979	0.030	0.613	−0.015	0.858
Humanized Educational Experience Sum Score	−0.025	0.541	−0.073	0.364	0.037	0.522	−0.042	0.602
Holistic Support Sum Score	0.004	0.928	0.118	0.146	**−0.051**	**0.007**	0.120	0.133
Academic Progress Sum Score	−0.025	0.560	−0.116	0.146	−0.013	0.819	0.006	0.944
Social Support (Family) Sum Score	−0.060	0.151	0.042	0.606	−0.099	0.081	−0.056	0.499
Social Support (Friends) Sum Score	−0.019	0.631	0.142	0.073	−0.056	0.347	−0.092	0.245
Age	**0.027**	**0.026**	0.054	0.509	0.079	0.154	0.055	0.497
BMI	**0.018**	**0.009**	0.010	0.905	**0.030**	**0.017**	**0.053**	**0.019**
Sex (Female)	0.006	0.873	0.036	0.647	0.047	0.399	0.005	0.953
Race (Black)	**0.301**	**0.003**	-	-	-	-	-	-
Ethnicity (Hispanic)	0.003	0.961	-	-	-	-	-	-
Currently Employed	−0.034	0.571	0.113	0.156	0.042	0.452	−0.075	0.353
Parents/Family Financially Support	−0.046	0.266	0.074	0.359	−0.059	0.290	−0.114	0.156
Loans Financially support	−0.038	0.340	−0.036	0.653	0.028	0.608	−0.133	0.103
Grants/Scholarships Financial Support	−0.003	0.944	0.021	0.790	−0.017	0.752	−0.034	0.675
Working to Financially Support	**−0.205**	**0.038**	−0.118	0.137	−0.058	0.305	0.048	0.554
Pell Grant	0.008	0.849	0.005	0.955	−0.010	0.856	0.132	0.095
Meal Plan	−0.042	0.325	0.070	0.380	−0.097	0.087	0.025	0.757
Disability	−0.021	0.605	0.025	0.758	−0.080	0.148	0.038	0.635
Health Status (Poor/Fair)	0.049	0.251	0.031	0.704	0.028	0.631	0.150	0.068
Cooking for Self/Others	0.075	0.079	0.072	0.369	0.039	0.508	0.088	0.289
Perceived Cooking Skills	0.011	0.780	−0.002	0.978	0.027	0.634	0.053	0.519
Enrollment (FT/PT)	−0.001	0.982	0.064	0.423	0.015	0.783	0.003	0.966
Freshman	0.010	0.809	0.150	0.061	−0.063	0.252	−0.012	0.877
Sophomore	0.029	0.475	−0.003	0.970	−0.028	0.617	0.035	0.664
Junior	−0.038	0.346	−0.055	0.495	0.016	0.776	−0.115	0.160
Senior	0.007	0.875	−0.059	0.456	0.062	0.272	0.090	0.263
Campus/University Housing	−0.031	0.463	0.068	0.394	−0.072	0.200	0.015	0.856
Living with Parent/Guardian	−0.037	0.366	−0.058	0.467	−0.019	0.741	−0.068	0.392
Off Campus/Non-University Housing	0.054	0.187	−0.029	0.721	0.071	0.216	0.064	0.425

Note: Statistically significant values (*p* < 0.05) and corresponding coefficients are bolded. Abbreviations: BMI, body mass index; FI, food insecurity; FT, full-time; PT, part-time. Adjusted R^2^: Model 1 = 0.208; Model 2 = 0.078; Model 3 = 0.240; Model 4 = 0.323.

**Table 6:** Highlights of key study findings.

No statistically significant differences were observed for FI by racial and ethnic group.
Regardless of race or ethnicity, working ≥ 20 h per week to financially support oneself and race (Black) are among the strongest predictors of FI among college students.
Discrimination major was the sole predictor of FI for non-Hispanic Black students.
Coping mechanisms for FI (savings, reduced intake) and BMI were predictors of FI for Hispanic and non-Hispanic White students.
Decreased holistic support from faculty and staff was observed as a predictor of FI in Hispanic students.

## Data Availability

Data can be provided upon request from the authors.
